# Fish oil mitigates myosteatosis and improves chemotherapy efficacy in a preclinical model of colon cancer

**DOI:** 10.1371/journal.pone.0183576

**Published:** 2017-08-23

**Authors:** Alaa A. Almasud, Kaitlin H. Giles, John J. Miklavcic, Karen J. B. Martins, Vickie E. Baracos, Charles T. Putman, Leluo L. Guan, Vera C. Mazurak

**Affiliations:** 1 Alberta Institute for Human Nutrition, Department of Agricultural, Food and Nutritional Sciences, University of Alberta, Edmonton, AB, Canada; 2 Palliative Care Medicine, Department of Oncology, University of Alberta, Edmonton, AB, Canada; 3 Exercise Biochemistry Laboratory, Faculty of Physical Education and Recreation; Neuroscience and Mental Health Institute, Faculty of Medicine and Dentistry, University of Alberta, Edmonton, AB, Canada; 4 Functional Genomics and Microbiology, Department of Agricultural, Food and Nutritional Science, University of Alberta, Edmonton, AB, Canada; Max Delbrueck Center for Molecular Medicine, GERMANY

## Abstract

**Background:**

This study aimed to assess whether feeding a diet containing fish oil was efficacious in reducing tumor- and subsequent chemotherapy-associated myosteatosis, and improving tumor response to treatment.

**Methods:**

Female Fischer 344 rats were fed either a control diet for the entire study (control), or switched to a diet containing fish oil (2.0 g /100 g of diet) one week prior to tumor implantation (long term fish oil) or at the start of chemotherapy (adjuvant fish oil). Chemotherapy (irinotecan plus 5-fluorouracil) was initiated 2 weeks after tumor implantation (cycle-1) and 1 week thereafter (cycle-2). Reference animals received no tumor or treatment and only consumed the control diet. All skeletal muscle measures were conducted in the gastrocnemius. To assess myosteatosis, lipids were assessed histologically by Oil Red O staining and total triglyceride content was quantified by gas chromatography. Expression of adipogenic transcription factors were assessed at the mRNA level by real-time RT-PCR.

**Results:**

Feeding a diet containing fish oil significantly reduced tumor- and subsequent chemotherapy-associated increases in skeletal muscle neutral lipid (p<0.001) and total triglyceride content (p<0.03), and expression of adipogenic transcription factors (p<0.01) compared with control diet fed animals. The adjuvant fish oil diet was as effective as the long term fish oil diet in mitigating chemotherapy-associated skeletal muscle fat content, and in reducing tumor volume during chemotherapy compared with control fed animals (p<0.01).

**Conclusion:**

Long term and adjuvant fish oil diets are equally efficacious in reducing chemotherapy-associated myosteatosis that may be occurring by reducing expression of transcription factors involved in adipogenesis/lipogenesis, and improving tumor-response to chemotherapy in a neoplastic model.

## Introduction

Clinical practice aims to improve the effectiveness of chemotherapeutics to reduce tumor growth while mitigating harmful side effects. One approach to improve the therapeutic index of antineoplastic therapies is to combine cytotoxic drugs with adjuvant factors that enhance anti-tumor efficacy and reduce harmful side effects. Pathological alterations in skeletal muscle have been identified in cancer patients undergoing treatment, including muscle loss and fat accumulation [1–7]. Specifically, myosteatosis, defined as the pathological accumulation of fat in skeletal muscle, is emerging as an important prognostic factor in the oncology setting [2,4–6]. Low skeletal muscle density, which reflects high skeletal muscle fatty infiltration, is associated with shorter progression- and disease-free survival [2,6], and overall survival [2,4,5] in cancer patients treated with various therapies including chemotherapy. Therefore, identification of an adjuvant factor to chemotherapy that can protect against myosteatosis, in addition to enhancing tumor cytotoxicity, would be of great benefit.

The omega-3 polyunsaturated fatty acids, eicosapentaenoic acid [EPA, 20:5n-3] and docosahexaenoic acid [DHA, 22:6n-3], which are highly abundant in fish oil, are emerging as promising nutritional adjuvants to chemotherapy as they have been reported to reduce drug-associated toxicities and enhance anti-tumor effects in a variety of antineoplastic agents in a number of *in vitro* and *in vivo* preclinical models (reviewed by [8]). There is also accumulating evidence from clinical trials that EPA and DHA supplementation improves patient outcomes during cancer chemotherapy, including improved skeletal muscle condition and a greater tumor response rate (reviewed by [9]). Specifically, we showed that advanced non-small cell lung cancer patients who supplemented with EPA and DHA during treatment had a preservation of skeletal muscle mass, less intermuscular adipose tissue, and better tumor responses compared to those not taking fish oil (standard of care) [10,11]. Therefore, EPA and DHA may protect against myosteatosis while improving tumor response to antineoplastic agents. While EPA and DHA have been shown to improve myosteatosis in other human pathological conditions (reviewed by [12]), it has yet to be investigated in the oncology setting. Appropriate preclinical models are required to investigate the biological features and causes of cancer-associated myosteatosis, as well as the mechanisms through which EPA and DHA may be exerting their protective effects in the tumor-bearing state, with and without chemotherapy.

While conditions such as insulin resistance and obesity suggest that impaired skeletal muscle fatty acid metabolism may be responsible for pathological fat accumulation in muscle [13], cancer-associated myosteatosis may also involve mechanisms related to adipogenesis. Specifically, cancer has been shown to upregulate the expression of adipogenic genes in skeletal muscle, including CCAAT/enhancer-binding protein (*C/EBP*)*β*, a potent activator of adipogenesis [14]. *C/EBPβ*, *δ*, and *α*, and peroxisome proliferator-activated receptor (*PPAR*)*γ*, are important transcription factors involved in robust adipocyte gene expression [15]. For example, overexpression of *C/EBPα* and/or *PPARγ* have been shown to convert myoblasts into adipocytes by promoting adipogenesis and lipogenesis [16], and increase skeletal muscle triglyceride (TG) *in vivo* [17]. Reducing the n-6/n-3 ratio decreased the expression of *PPARγ* and inhibited adipogenesis in the 3T3-L1 pre-adipocyte cell line [18]. *In vivo*, feeding a diet rich in fish oil prevented myosteatosis (induced by a lard-based high fat diet) with no changes in fatty acid oxidation, suggesting an alternative mechanism by which n-3 fatty acids modify the fat content of skeletal muscle [19]. Collectively, it appears that cancer-associated myosteatosis may involve mechanisms related to adipocyte gene expression, which can potentially be modified in the presence of n-3 fatty acid supplementation.

We have established an animal model to study interactions amongst tumor (rats bearing the Ward colon tumor) and chemotherapy, combined irinotecan (CPT-11) and 5-fluorouracil (5-FU) [20,21] that represents a first-line chemotherapy treatment regime for colorectal cancer, and elicits a similar level of toxicity as observed in humans treated with this drug combination. The current study aimed to identify a preclinical model of cancer-associated myosteatosis and then, apply this model to investigate the effects of dietary EPA and DHA on tumor- and subsequent chemotherapy-associated fat content of skeletal muscle, as well as the tumor response to chemotherapy. Additionally, we wanted to determine whether feeding dietary EPA and DHA beginning at the initiation of chemotherapy (adjuvant) was able to elicit similar effects compared to a diet fed beginning prior to tumor implantation (long term). We hypothesized that EPA and DHA supplementation would prevent tumor-associated myosteatosis before treatment, and that both the long term and adjuvant fish oil diets would similarly mitigate chemotherapy-associated fat accumulation in skeletal muscle through the inhibition of adipogenic/lipogenic transcription factor signaling, as well as enhance the tumor response to chemotherapy.

## Materials and methods

Experimental procedures were reviewed and approved by the University of Alberta Institutional Animal Care Committee and conducted in accordance with the Guidelines of the Canadian Council on Animal Care.

### Animal model and experimental design

Female Fischer 344 rats (n = 72) weighing an average of 127 ± 18 g aged 11–12 weeks were received from Charles River (St. Constant, QC, Canada). Vendor health reports indicated that the colony from which rats were obtained were free of known viral and parasitic pathogens; klebsiella pneumoniae, streptococcus and staphylococcus aureus bacterial pathogens were detected in the colony. Rats were housed two per cage containing bedding, tubing and balls for environmental enrichment, and covered with and filter tops during the seven-day acclimation period and one per cage when initial diets were assigned. Rats received twelve hours of a light:dark cycle per day, and were kept in a positive air pressure room at a constant temperature (22°C). Water and food was provided *ad libitum* throughout the entire experiment. All animal procedures were conducted during the light cycle in the animal facility.

**Fig 1:**
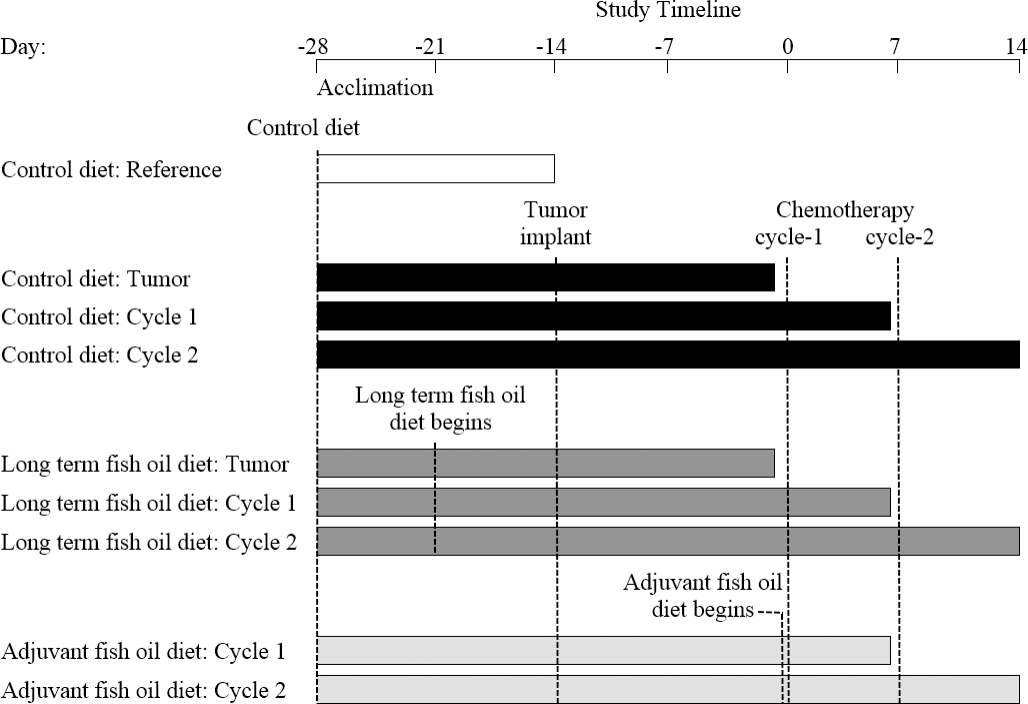
Experimental study design. Bars show the length of time each group was in the study. Dashed lines show when indicated interventions occurred.

Experimental design is outlined in Fig 1. All rats were initially fed a control diet during the seven-day acclimation period and then, one week prior to tumor implantation, rats were randomly assigned to one of three diets by picking group assignment out of a hat: 1) control diet (n = 24); 2) long term fish oil diet (n = 24); 3) adjuvant fish oil diet (control diet until chemotherapy was initiated, then switched to the fish oil diet; n = 16).

To examine a potential preclinical model of cancer-associated myosteatosis, two weeks after tumor implantation, rats on the control diet were either euthanized (n = 8) or underwent one cycle (cycle-1; n = 8) or two cycles (cycle-2; n = 8) of chemotherapy. To investigate the effects of EPA and DHA supplementation before and during chemotherapy treatment, a group of rats on the long term fish oil diet were euthanized two weeks after tumor implantation (n = 8), while the remaining rats on the long term and adjuvant fish oil diets underwent one cycle (n = 8 each) or two cycles (n = 8 each) of chemotherapy. Rats serving as a reference group (n = 8) did not undergo tumor implantation or receive chemotherapy, consumed only the control diet throughout the entire study, and were otherwise handled in the same manner as the experimental groups.

### Tumor injection and chemotherapy

The Ward colorectal primary carcinoma (0.05 g; provided by Dr. Y Rustum, Roswell Park Institute Buffalo, NY, USA) was transplanted subcutaneously into the flank of the rats under mild isoflurane anesthesia. Tumor size was calculated as described previously [20,21]. Tumor volume was recorded every other day prior to initiation of chemotherapy, and every day during the two weeks that chemotherapy was administered. During chemotherapy, relative tumor volume for each animal was compared to the baseline volume (Day 0).

The day when chemotherapy was initiated was designated as Day 0. Cycle-1 consisted of CPT-11 (50 mg/kg body weight, *intraperitoneal*) administered on Day 0 and 5-FU (50 mg/kg body weight, *intraperitoneal*) administered on Day 1. Cycle-2 consisted of the same drug regime occurring one week after cycle-1 (Days 7 and 8, respectively). Atropine (1 mg/kg body weight, *subcutaneous*) was administered immediately prior to each CPT-11 injection to alleviate early onset cholinergic symptoms [20].

### Diet and food intake

Animals were fed a basal diet (Teklad TD.84172 basal mix with fat source omitted, Harlan Teklad, Madison, WI) with added fat (20 g/100 g; Table 1). Different mixtures of commercially available fats (i.e. canola stearine, olive oil, sunflower oil and canola oil with or without fish oil (Ocean Nutrition Canada, Dartmouth, NS, Canada)) were added to the diets to ensure that the saturated, monounsaturated, and polyunsatured contents were similar between the control and fish oil diets, and differed only in their EPA and DHA contents. Both diets contained 40% of total energy from fat, 40% from carbohydrates and 20% from protein, representing the estimated average proportion of macronutrients typically consumed by humans. Food intake was measured every other day prior to initiation of chemotherapy, and every day during the two weeks that chemotherapy was administered. During chemotherapy, relative food intake for each animal was compared to the average relative food intake prior to chemotherapy (Day -14 to Day 0).

**Table 1:** Composition of experimental diets.

Ingredient	Control diet	Fish oil diet
***Constant portion (80% w/w of diet)***		
Basal mix (g/100 g of diet):		
Casein	33.8 g	33.8 g
L-Methionine	0.3 g	0.3 g
Dextrose, monohydrate	26.1 g	26.1 g
Corn starch	25.0 g	25.0 g
Vitamin mix, AOAC (40055)	1.3 g	1.3 g
Mineral mix, Bemhart-Tomarelli (170750)	6.4 g	6.4 g
Inositol	0.8 g	0.8 g
Choline chloride	0.2 g	0.2 g
Cellulose	6.3 g	6.3 g
Sodium selenite	0.04 g	0.04 g
Manganese sulfate, monohydrate	0.03 g	0.03 g
***Variable portion (20% w/w of diet)***		
Lipid source (g/100 g of diet):		
Canola stearine	11.7 g	12.0 g
Olive oil	0.0 g	0.8 g
Sunflower oil	5.2 g	3.6 g
Canola oil	3.1 g	1.6 g
Fish oil	0.0 g	2.0 g
Fatty acid composition (% of total fatty acids in the lipid source):		
Saturated fatty acids	58.7%	59.9%
Monounsaturated fatty acids	17.3%	14.3%
Polyunsaturated fatty acids	20.6%	22.5%
Total n-6	18.6%	13.6%
Total n-3	2.0%	8.9%
EPA	0.0%	5.1%
DHA	0.0%	2.1%

Diets were isocaloric and isonitrogenous. AOAC, Association of Official Agricultural Chemists; DHA, docosahexaenoic acid; EPA, eicosapentaenoic acid. Fatty acid composition was measured by gas chromatography.

### Body weight

Body weight was recorded on the same days as tumor volume. Body weight was converted to tumor-free body weight for data interpretation and statistical analysis. Body weight during chemotherapy was expressed relative to each animal’s body weight at Day 0.

### Study termination and tissue collection

All rats were euthanized by carbon dioxide (CO_2_) asphyxiation. At euthanization, gastrocnemius muscles were isolated, weighed, and frozen in melting isopentane cooled in liquid nitrogen (-156°C), and stored at -80°C until subsequent analyses.

### Oil Red O and hematoxylin staining

Frozen gastrocnemius muscles were cryosectioned transversely (10 μm thick) and stained for neutral lipid content using Oil Red O as previously described [22]. Sections were then rinsed in distilled water, and counterstained (5 min) in Mayer’s Hematoxylin (Sigma-Aldrich, St. Louis, MO, USA), to delineate fibers for cross-sectional area (CSA) measurements, before another rinse in distilled water. Sections were visualized under a ZEISS AXIO Compound Light Microscope (AX10 Scope A.1, Carl Zeiss Group, Toronto, ON, Canada) at 200× magnification. Colour images were taken with an Optronics MacroFire Digital Camera (Optronics, Goleta, CA, USA) using a Leica TCS-SP2 spectral confocal and multiphoton system (Leica Camera, Solms, Germany). Qualitative and quantitative analysis of Oil Red O staining was performed in a blinded manner and analyzed using Volocity 6.3 software (PerkinElmer, Inc., Waltham, MA, USA). An average of 800 ± 23 fibers were analyzed per muscle for quantification of fibers expressing Oil Red O staining. Skeletal muscle fiber CSA was measured using Image J software on 200 fibers per muscle.

### Fatty acid and triglyceride quantification and composition

Gastrocnemius muscle (100 mg) was homogenized in a 1.6 ml calcium chloride (CaCl_2_; 0.025%) solution with glass beads (0.5 mm diameter; FastPrep ®-24, MP Biomedicals, Santa Ana, CA, USA) in 20 sec intervals for 1 min total. Samples were placed on ice for at least 15 sec between each homogenization interval. Lipids were extracted using chloroform/methanol as previously described [23]. The TG fraction was isolated on G-plates as previously described [24,25]. The TG band was scraped from G-plates and the C15:0 internal standard (10.2 mg/100 ml hexane) was added, followed by saponification. TG was then methylated as previously described [25]. Fatty acid composition was determined using gas chromatography-flame-ionisation detector analysis on a Varian 3900 (Varian Instruments, Georgetown, ON, Canada) as previously described [25]. Peaks of saturated, monounsaturated and polyunsaturated fatty acids were separated between 6 and 24 carbon chain lengths and identified using a fatty acid standard of known composition (GLC461, Sigma-Aldrich). Quantity of fatty acids within the TG fractions were calculated by comparison with the known concentration of the C15:0 standard.

### RNA extraction and quantitative real-time polymerase chain reaction (qRT-PCR)

Total RNA was extracted from gastrocnemius muscle (10 mg) using the MagMax-96 total RNA isolation Kit (Ambion, Austin, TX, USA) following the manufacturer’s protocol. For assessing RNA quantity and quality, a NanoDrop spectrophotometer (Thermo Scientific, Wilmington, DE) and Agilent 2100 Bioanalyzer (Agilent Technologies, Santa Clara, CA, USA) were used, respectively. Samples were then diluted with nuclease-free water to 7 ng/μl. A High Capacity cDNA Reverse Transcription kit (Applied Biosystems, Foster City, CA, USA) was used to reverse transcribe RNA to cDNA following the manufacturer’s protocol. Pre-designed TaqMan® probes with a 6-carboxyfluorescein phosphoramidite (FAM™) label on the 5' end and primer sets (Applied Biosystem) were used to target the following genes: *C/EBPβ* (Rn01764319_m1), *C/EBPδ* (Rn02532096_s1), *C/EBPα* (Rn00560963_s1), *PPARγ* (Rn00440945_m1), and *SREBP*-1c (Mm00550339_g1). *18S* rRNA (Rn03928990_g1) was stable among all samples, and therefore used as the endogenous control. qRT-PCR was performed on 1 μ*l* cDNA samples, in triplicate, on an ABI 7900HT thermocycler (Applied Biosystems). Relative changes in gene expression were determined using the 2^−ΔΔCT^ method of analysis [26].

### Statistical analysis

The primary outcome measure, fat accumulation in skeletal muscle was analyzed. Additionally, three secondary outcome measures were evaluated: adipogenic transcription factors, skeletal muscle weight and cross-sectional area, and the tumor response to chemotherapy.

Data are summarized as mean ± SD. One-way and a two-way repeated measures analysis of variance (ANOVA) were used to test differences in food intake, changes in body weight and tumor volume before and during chemotherapy treatment, respectively. A One-way ANOVA was used to test differences in Oil Red O staining, total TG content, mRNA fold changes in gene expression, and fatty acid content. When a significant difference was observed, either a post-hoc analysis was completed using the Bonferroni model or planned comparisons were performed on data in which *a priori* hypotheses were established. A Pearson’s correlation was used to test relationships between mRNA expression of various adipogenic/lipogenic transcription factors and total TG content. Statistical significance was reported when *p* value <0.05. All statistical analyses were performed using SPSS 21.0 (Chicago, IL, USA) for Windows.

## Results

### General animal health

Of the 72 female Fischer 344 rats used in this study, one rat died one day before planned euthanization (control diet, cycle 2).

### Food intake and body weight

After tumor implantation and prior to chemotherapy, the rats consuming the fish oil diet had a significantly higher relative food intake compared to the rats consuming the control diet (1.0 ± 0.1 g/day versus 0.9 ± 0.1 g/day, p<0.001; data not shown). During this time, rats consuming the fish oil diet gained 13 ± 5 g body weight compared to 7 ± 3 g in the rats consuming the control diet (p< 0.03).

After each cycle of chemotherapy, relative food intake initially decreased in all groups, but returned to baseline by the end of the cycle; there were no significant differences between the groups (0.8 ± 0.1 g/day; data not shown). Relative body weight decreased after each chemotherapy cycle and was significantly lower than baseline on the second, third and fourth day following chemotherapy injection (body weight decreased by 5%; p = 0.04). Average intake of EPA plus DHA during the chemotherapy period in the fish oil groups was 112 ± 25 mg/day.

### Identification of a potential model of tumor- and chemotherapy-associated myosteatosis

#### Fat accumulation in skeletal muscle

**Fig 2:**
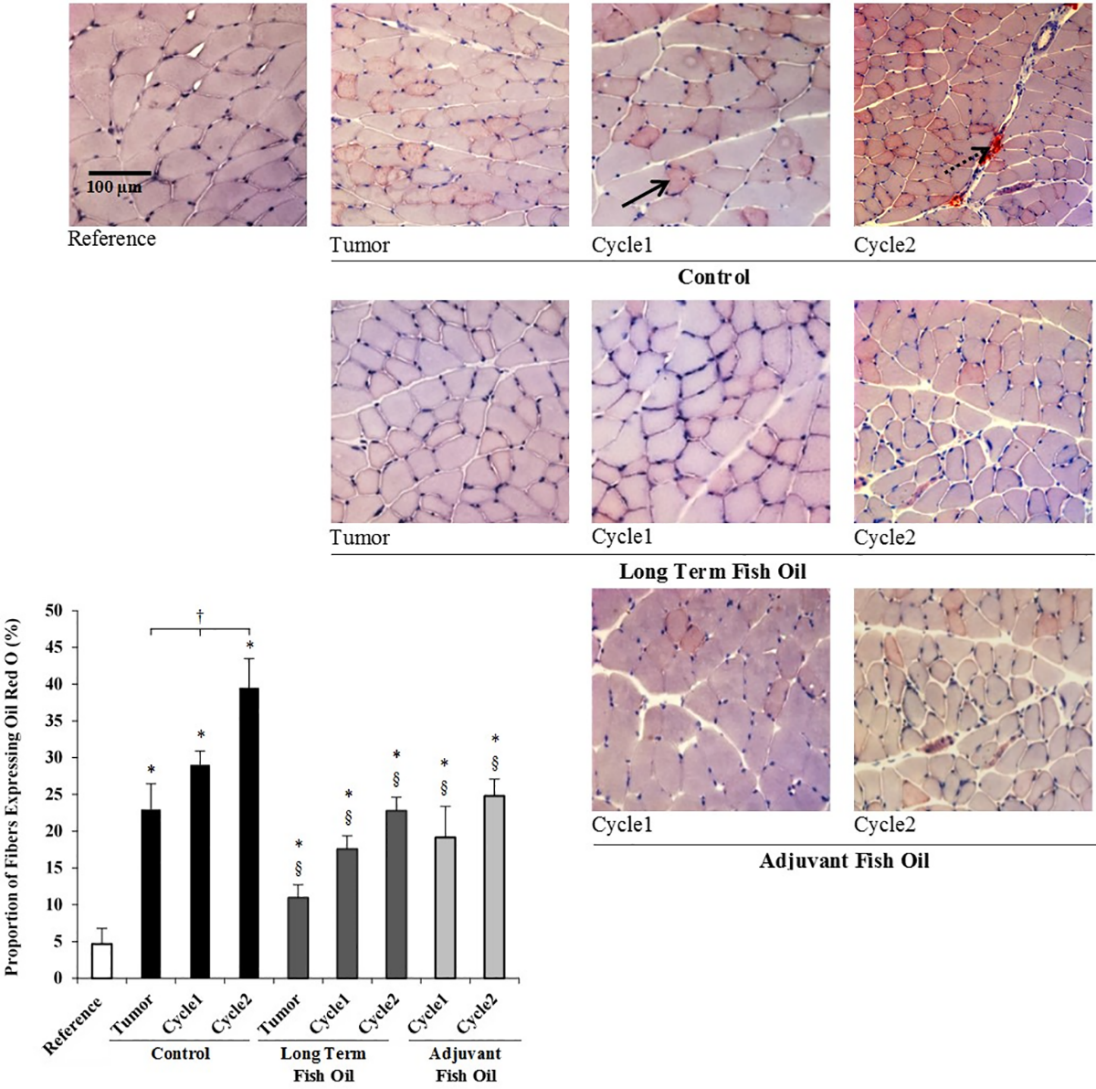
Oil Red O staining and analysis of neutral lipid localization in rat gastrocnemius muscle. (A) Representative photomicrographs of Oil Red O stained skeletal muscle. Solid arrow shows an example of a positive fiber expressing Oil Red O; dashed arrow shows an example of Oil Red O staining between muscle fibers. Scale bar represents 100 μm. (B) Proportion of positive fibers expressing Oil Red O staining. Values are means ± SD. Statistical symbols indicate: *different from Reference; ^†^different amongst the indicated control diet groups; ^§^different from relative control diet group (p<0.05).

**Fig 3:**
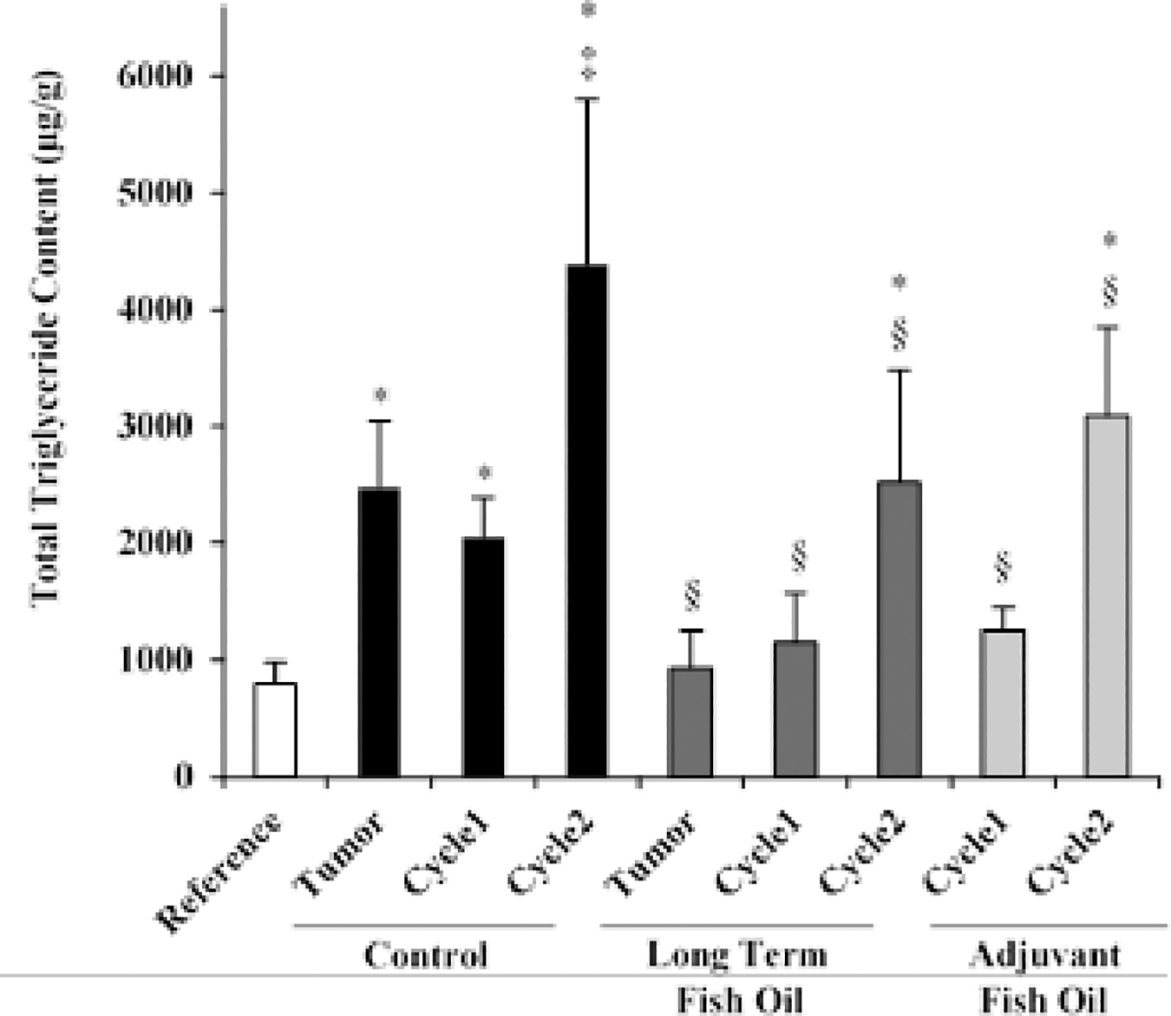
Total triglyceride content in the rat gastrocnemius muscle quantified using gas chromatography. Values are means ± SD. Statistical symbols indicate: *different from Reference; ^‡^different from tumor-bearing and cycle-1 control diet groups; ^§^different from relative control diet group (p<0.05).

First, to confirm that the Ward colon tumor-bearing rat and CPT-11/5-FU delivery models induced skeletal muscle fat accumulation, we quantified both the neutral lipid accumulation and total TG content in the gastrocnemius muscle of rats on the control diet. Neutral lipids were quantified according to strong positive staining for Oil Red O within skeletal muscle fibers, and qualitatively for the presence of intermuscular fiber variations (Fig 2). Two weeks following tumor implantation, the proportion of fibers expressing Oil Red O and the total TG content in the gastrocnemius muscle were 5-times (p<0.001, Fig 2) and 3-times (p<0.001, Fig 3) higher, respectively, in the control diet group compared with the reference group, reflecting tumor-associated fat accumulation in skeletal muscle. Subsequently, chemotherapy treatment further increased fat accumulation in the gastrocnemius muscle. Rats on the control diet who underwent 2 cycles of chemotherapy displayed a large amount of intermuscular fiber Oil Red O staining (Fig 2), and significant increases in the proportion of fibers expressing Oil Red O (Fig 2) and total TG content (Fig 3) compared with tumor-bearing only rats (p<0.001 and p<0.001, respectively) and rats who underwent cycle-1 (p<0.001 and p<0.001, respectively).

#### Adipogenic transcription factors in skeletal muscle

**Fig 4:**
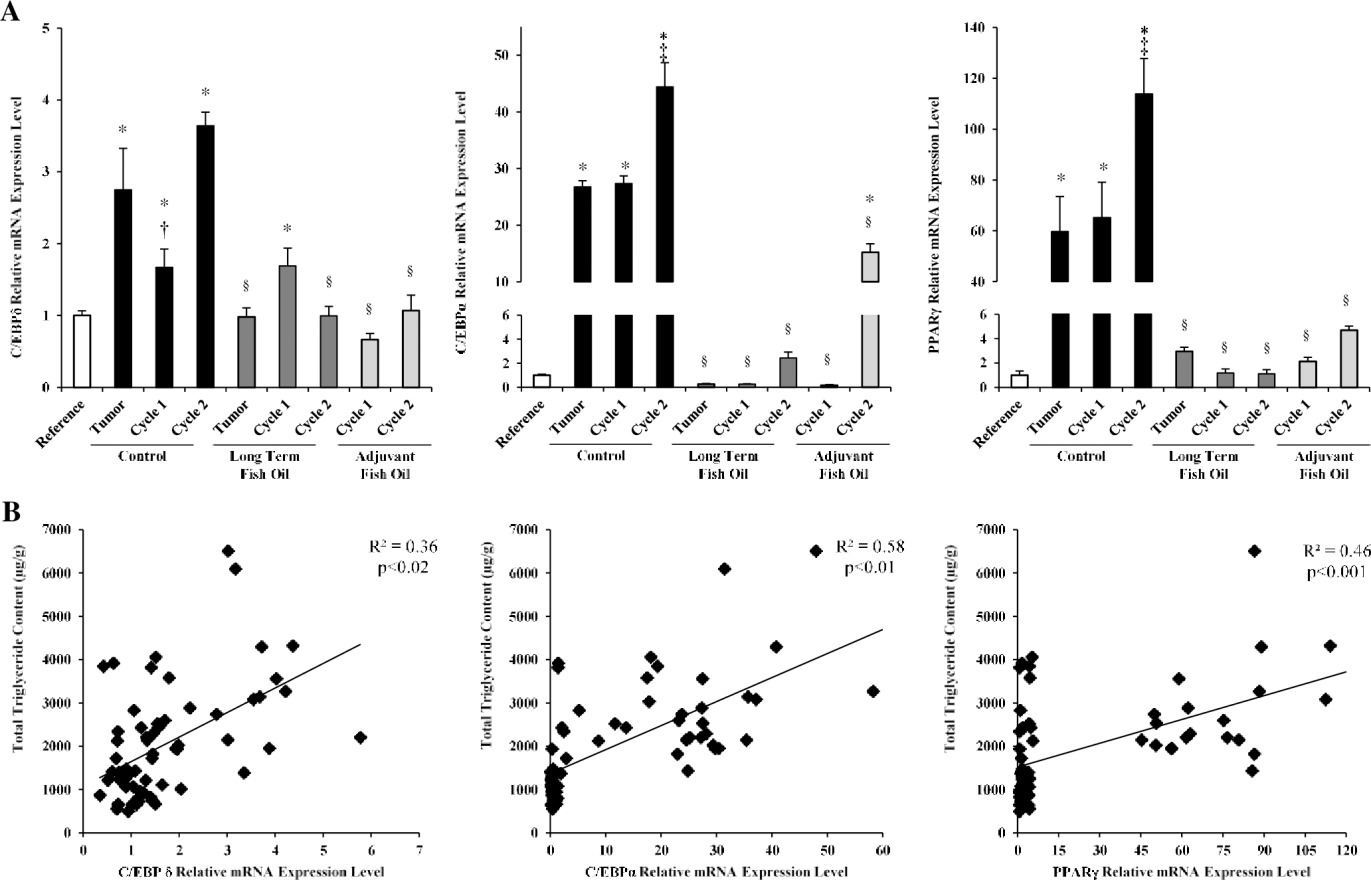
Patterns of adipogenic transcription factor mRNA expression in the rat gastrocnemius muscle. (A) Relative changes in *C/EPBδ*, *C/EBPα* and *PPARγ* mRNA expression levels as determined by the 2^−ΔΔCT^ method of analysis. Values are means ± SD. Statistical symbols indicate: *different from Reference; ^‡^different from tumor-bearing and cycle-1 control diet groups; ^†^different from tumor-bearing and cycle-2 control diet groups; ^§^different from relative control diet group (p<0.05). (B) The correlation between each gene and the total triglyceride content.

Next, we assessed expression of genes involved in the adipogenic transcription cascade including the upstream genes sterol regulatory element binding protein *(SREBP)-1c*, *C/EBPβ* and *C/EBPδ*, as well as the downstream genes *C/EBPα* and *PPARγ* in the gastrocnemius muscle of rats on the control diet to identify a potential mechanism for skeletal muscle fat accumulation in response to the tumor-bearing state before and during chemotherapy treatment. While expression of *SREBP-1c* and *C/EBPβ* were not altered (S1 Fig), *C/EBPδ* displayed a significant increase in response to the tumor-bearing state (p<0.001) and after each cycle of chemotherapy (cycle-1, p<0.05; cycle-2, p<0.001) in animals on the control diet compared with reference animals (Fig 4A). Expression of *C/EBPα* and *PPARγ* showed remarkable increases in response to the tumor-bearing state and subsequent chemotherapy treatment in rats on the control diet (Fig 4A). Specifically, in these animals, *C/EBPα* and *PPARγ* expression were 26-times (p<0.001) and 59-times (p<0.001) higher, respectively, in the tumor-bearing state compared with reference animals (Fig 4A). *C/EBPα* and *PPARγ* expression further significantly increased after cycle-2 compared with tumor-bearing (p<0.001 and p<0.001, respectively) and cycle-1 animals (p<0.001 and p<0.001, respectively) on the control diet.

We further explored the relationships between *C/EBPδ*, *C/EBPα* and *PPARγ* expression and total TG content in the gastrocnemius muscle (Fig 4B). The expression of *C/EBPδ*, *C/EBPα* and *PPARγ* were significantly positively correlated with total TG content (Fig 4B), revealing that key transcription factors involved in adipogenesis are related to total TG content in skeletal muscle.

#### Skeletal muscle weight and fiber cross-sectional area

Further characterization of the gastrocnemius muscle showed that while skeletal muscle weight was not different between all of the groups (overall mean 760 ± 60 mg; data not shown), the tumor-bearing state (p<0.02) and subsequent chemotherapy (p<0.001) resulted in significantly lower skeletal muscle fiber CSA in rats on the control diet compared with reference animals (S2 Fig). This shows that moderate skeletal muscle atrophy occurred concurrent with fat accumulation in this animal model.

### Effects of the fish oil diets on tumor- and chemotherapy-associated myosteatosis

#### EPA and DHA content in skeletal muscle

Since chemotherapy treatment significantly reduced the EPA (i.e. cycle-2) and DHA (i.e. cycle-1 and cycle-2) content in the gastrocnemius muscle TG fraction of rats on the control diet compared with the reference animals (Table 2), we wanted to first confirm that the fish oil diets restored skeletal muscle EPA and DHA. Indeed, both fish oil diets were efficacious in maintaining or elevating EPA and DHA throughout both cycles of chemotherapy compared to reference (Table 2).

**Table 2:** EPA and DHA content within the rat gastrocnemius muscle tryglyceride fraction.

n3-PUFA	Study Groups									P Value
	Control Diet				Long Term Fish Oil Diet			Adjuvant Fish Oil Diet		
	Reference	Tumor	Cycle1	Cycle2	Tumor	Cycle1	Cycle2	Cycle1	Cycle2	
EPA (μg/g)	0.9 ± 0.5^a^	0.4 ± 0.2^ab^	0.7 ± 0.5^a^	0.2 ± 0.2^b^	0.9 ± 0.4^a^	0.9 ± 0.1^a^	1.3 ± 0.7^c^	1.5 ± 0.3^c^	1.1 ± 0.5^ac^	p<0.001
DHA (μg/g)	0.7 ± 0.4^a^	0.6 ± 0.4^a^	0.1 ± 0.0^b^	ND^b^	1.7 ± 0.5^c^	2.6 ± 1.3^d^	2.6 ± 0.7^d^	2.7 ± 0.8^d^	1.7 ± 0.5^d^	p<0.001

Values are presented as means ± SD. Different letters indicate significant differences among groups. DHA, docosahexaenoic acid; EPA, eicosapentaenoic acid; ND, not detectable; PUFA, polyunsaturated fatty acid.

#### Fat accumulation in skeletal muscle

To test the hypothesis that in a Ward colon tumor-bearing rat model of myosteatosis, dietary EPA and DHA mitigate tumor- and chemotherapy-associated fat accumulation in skeletal muscle, we measured neutral lipids and total TG content in the gastrocnemius muscle of rats fed a diet containing fish oil beginning one week prior to tumor implantation (long term) and beginning when chemotherapy was initiated (adjuvant). Before chemotherapy, tumor-bearing rats on the long term fish oil diet displayed significantly lower proportions of fibers expressing Oil Red O (p<0.001; Fig 2) and total TG content (p<0.001; Fig 3) in the gastrocnemius muscle compared with tumor-bearing rats on the control diet. Subsequently, after both cycle-1 and cycle-2, tumor-bearing rats on the long term and adjuvant fish oil diets displayed minimal intermuscular Oil Red O staining (Fig 2), and similarly exhibited significantly less fibers expressing Oil Red O (p<0.001; Fig 2) and total TG content (p<0.03; Fig 3) in the gastrocnemius muscle compared with their respective chemotherapy treated cycle control diet groups. Collectively, these results show that before chemotherapy, the long term fish oil diet prevented tumor-associated TG accumulation, and the adjuvant fish oil diet was equally efficacious as the long term fish oil diet in mitigating chemotherapy-associated fat accumulation in skeletal muscle.

#### Adipogenic transcription factors in skeletal muscle

For the most part, the fish oil diets prevented (long term) and reversed (adjuvant) tumor- and chemotherapy-associated increases in *C/EBPδ*, *C/EBPα* and *PPARγ* expression in the gastrocnemius muscle, as the expression of these transcription factors were not different from the reference group and were significantly lower compared with their respective tumor-bearing and chemotherapy treated cycle control diet groups (p<0.005; Fig 4A). Although *C/EBPδ* expression in the long term fish oil group significantly increased to the same level as the control diet group after cycle-1 compared with the reference group (p<0.04), this increase was transient as *C/EBPδ* expression returned to reference levels and was significantly lower compared with the control diet group after cycle-2 (p<0,001). Also, while cycle-2 *C/EBPα* expression was significantly greater in animals on the adjuvant fish oil diet compared with reference animals (p<0.001), it remained significantly lower compared with animals on the control diet after cycle-2 (p<0.001). Collectively, these results show that both fish oil diets greatly reduced tumor- and/or chemotherapy-associated increases in the expression of key adipogenic transcription factors.

#### Fiber cross-sectional area

All groups on the long term fish oil diet had similar skeletal muscle fiber CSA compared with reference animals, and were significantly greater than their respective control diet groups following one and two cycles of chemotherapy (p<0.001 and p<0.02, respectively; S2 Fig). Additionally, skeletal muscle fiber CSA was not different between the long term fish oil and adjuvant fish oil groups after either cycle-1 or cycle-2 (S2 Fig).

#### Effects of the fish oil diets on tumor response to chemotherapy

**Fig 5:**
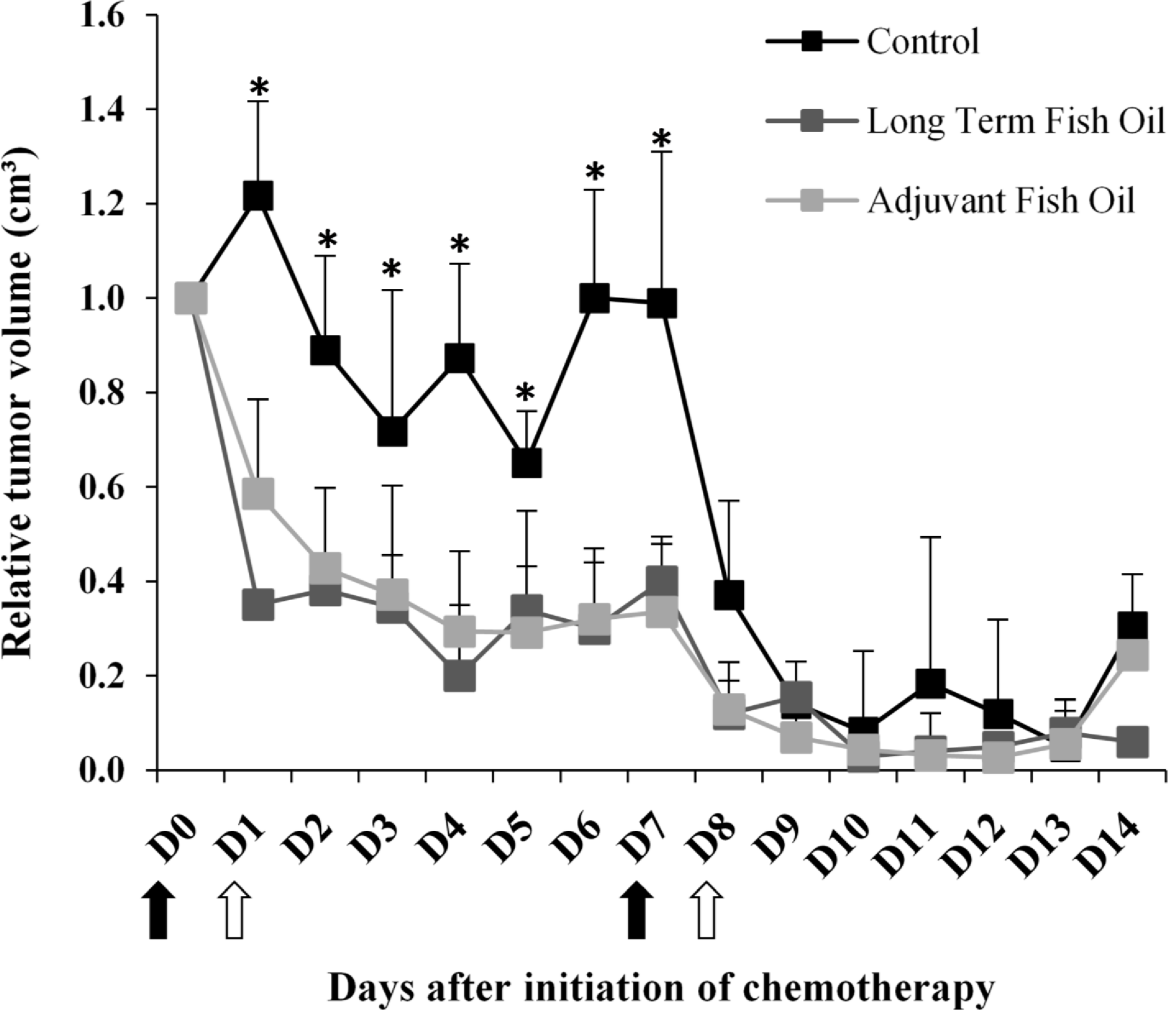
Relative tumor volume of tumor-bearing rats who underwent one or two cycles of CPT-11/5-FU treatment. Relative tumor volume was compared to the baseline volume when chemotherapy was initiated (Day 0). Black arrows show the days when single CPT-11 injections (50 mg/kg) occurred (Days 0, 7); white arrows show the days when single 5-FU injections (50 mg/kg) occurred (Days 1, 8). Values are means ± SD. *Indicates significant differences between the control and each fish oil diet group at each time point (p<0.01).

Tumors grew to 1.8 ± 0.4 cm^3^ in size and were similar between all groups before starting chemotherapy treatment. Long term and adjuvant EPA and DHA supplementation similarly enhanced the anti-tumor activity of CPT-11/5-FU chemotherapy compared with the control diet (Fig 5). In each of the 7 days following initiation of cycle-1, rats in both the long term and adjuvant fish oil groups had significantly smaller tumor volumes compared with tumor-bearing rats in the control diet group (p<0.01). Notably, tumor volumes were not significantly different between the long term and adjuvant fish oil diet groups during either cycle-1 or cycle-2.

## Discussion

To our knowledge, this study is the first to describe a preclinical model of tumor- and chemotherapy-associated myosteatosis. Using this model, we investigated the effects of a physiologically attainable level of EPA and DHA supplementation on the tumor-bearing state before and during chemotherapy-associated myosteatosis, as well as on the tumor response to chemotherapy. We show for the first time that feeding a diet containing EPA and DHA prevents tumor-associated myosteatosis, and that adjuvant supplementation is similarly efficacious as compared to long term EPA and DHA feeding in greatly mitigating chemotherapy-associated myosteatosis, and in enhancing the tumor response to chemotherapy. Specifically, we show that in the gastrocnemius muscle of tumor-bearing animals before and during chemotherapy, neutral lipids and total TG content were elevated along with increased expression of key transcription factors involved in adipocyte gene expression, which were all greatly reduced in animals fed a diet containing fish oil either beginning before tumor-implantation (long term) or at the initiation of chemotherapy (adjuvant). Additionally, after cycle-1, tumor volume decreased in animals on the long term and adjuvant fish oil diets, to an equal extent, compared with those on a control diet. Collectively, our results suggest that EPA and DHA supplementation is an effective adjuvant to chemotherapy for combating myosteatosis, in addition to its well-documented effect of enhancing tumor cytotoxicity (reviewed by [8]).

Myosteatosis is emerging as an important prognostic factor in the oncology setting, as it has been found to be associated with shorter survival in cancer patients treated with various therapies including chemotherapy [2,4–6]. To our knowledge, preclinical tumor-bearing models of myosteatosis have not been reported. In this study, the Ward colon tumor model displayed robust fat accumulation within skeletal muscle that was exacerbated by successive chemotherapy cycles. Rollins *et al*. [5] has reported that the majority of cancer patients display myosteatosis in concert with skeletal muscle loss; two features that share a poor prognosis in advanced cachexic cancer patients [4]. Similarly, the Ward colon tumor model also displayed decreased skeletal muscle fiber cross-sectional area. These pathological changes in skeletal muscle have been identified during chemotherapy [3–5,7,25] and represent one of the toxicity effects of antineoplastic treatment. Collectively, this preclinical model displays features of human cancer with regards to skeletal muscle changes that occur in the majority of cancer patients undergoing chemotherapy.

Determining the anatomical location of pathological fat in cancer patients can be informative towards the identification of potential underlying signaling mechanisms. However, there is a dearth of information on this topic. Stephens *et al*. [27] examined intramuscular lipid droplets in weight-losing cancer patients, and their results suggest that the number and size of lipid droplets increase with cancer-associated weight loss. Similarly, we observed an increase in neutral lipid content primarily within skeletal muscle fibers, indicative of lipid droplets, in the tumor-bearing state that was exacerbated by successive chemotherapy cycles. Neutral lipids were also evident between skeletal muscle fibers, but only after the second cycle of chemotherapy, which may be attributed to the formation of intermuscular adipocytes. Collectively, it appears that signaling mechanisms involved in lipid droplet and adipocyte formation may be involved in cancer-associated myosteatosis.

Lipid droplet formation occurs in various tissues in response to cellular stress [28], which can occur via *SREBP-1c* [29], an adipogenic transcription factor that plays a key role in fatty acid biosynthesis [30]. Additionally, cellular stress responses occur in cancer and cancer therapies (reviewed by [31]). Therefore, we examined skeletal muscle *SREBP-1c* expression, but found that it was not altered in response to the tumor-bearing state or subsequent chemotherapy. However, *C/EBPδ*, *C/EPBα* and *PPARγ* expression, which were all elevated in the skeletal muscles of tumor-bearing animals before and after chemotherapy in our study, are involved in both lipogenesis and adipogenesis in skeletal muscle [16,32,33]. Specifically, increased *C/EBPδ* expression occurs in myopathic murine skeletal muscle that contains abnormally high amounts of lipid droplets within muscle fibers along with intermuscular adipocytes [32]. Additionally, *C/EBPα* and/or *PPARγ* overexpression *in vitro* increases fatty acid uptake and incorporation into skeletal muscle lipids including TG, and augments lipid droplet accumulation [16,33]. Likewise, these key transcription factors were positively correlated to skeletal muscle TG content in this animal model. Collectively, it appears that the mechanisms responsible for cancer-associated myosteatosis may involve key regulators of adipogenesis/lipogenesis.

Nutrient deficiencies have been observed in cancer patients undergoing chemotherapy including low levels of plasma EPA and DHA that appear to occur concurrently with skeletal muscle loss and fat deposition, which can be corrected when fish oil is provided to patients during treatment [10,26]. We showed that patients receiving first-line chemotherapy for advanced non-small cell lung cancer lost skeletal muscle mass and gained intermuscular fat, while patients who supplemented with EPA and DHA (2.1 g/day) beginning on the first day of chemotherapy exhibited a maintenance or gain in skeletal muscle mass and reduction in intermuscular fat over the same time period [10]. However, the mechanisms through which EPA and DHA supplementation exert this beneficial effect are unknown. Also, the extent to which adjuvant EPA and DHA supplementation during chemotherapy is as efficacious as beginning prior to a cancer diagnosis is important for application in the clinical setting.

EPA and DHA are known to have anti-adipogenic effects on adipose tissue and metabolism that occurs through multiple mechanisms [34], but little is known about this mechanistic regulation in cancer-associated fat accumulation in skeletal muscle. Here we demonstrate, for the first time, that EPA and DHA supplementation prevents tumor-associated increases in the transcription expression of *C/EBPδ*, *C/EBPα* and *PPARγ*, and greatly mitigates further increases during chemotherapy. These findings reveal that EPA and DHA appear to exert their beneficial effects, at least in part, through the suppression of key adipogenic transcription factors that we show are related to total TG content in skeletal muscle. Additionally, findings from our study also demonstrate that adjuvant feeding of EPA and DHA is as effective as long term feeding in mitigating myosteatosis and inhibiting the expression of key adipogenic/lipogenic transcription factors.

Our results identify a relevant preclinical model for the study of concurrent chemotherapy and myosteatosis. This research also contributes to the large body of preclinical (reveiwed by [8]) and emerging clinical [10,35] evidence suggesting that providing EPA and DHA concurrent with antineoplastic agents enhances anti-tumor effects. We additionally demonstrate that adjuvant is as effective as long term EPA and DHA supplementation in enhancing the anti-tumor activity of CPT-11/5-FU chemotherapy. These effects may be due to the incorporation of EPA and DHA in to cancer cell membrane phospholipids, thus modifying tumor properties. In doing so, a wide range of biological functions can be altered, such as eicosanoid production, signal transduction, membrane fluidity and cell interaction (reviewed by [36,37]). A limitation to this study is that animals were not pair-fed. Development of myosteatosis in the tumor-bearing state occurred in the absence of changes in food intake, and the decline in food intake that occurred following chemotherapy was restored within 3 days. Therefore, the marked changes observed were not attributable to changes in the food intake.

## Conclusions

The novel observations of pathological fat accumulation coupled with the increased expression of key adipogenic/lipogenic transcription factors in the skeletal muscles of an *in vivo* tumor-bearing model that underwent chemotherapy treatment, reveal a potential mechanism underlying fundamental alterations that may be involved in the development of myosteatosis in cancer patients. Long term and adjuvant dietary EPA and DHA feeding were equally efficacious in markedly improving the therapeutic index of CPT-11/5-FU chemotherapy by concurrently enhancing drug efficacy to the tumor while reducing the toxicity effect of fat accumulation within skeletal muscle. Our study highlights the therapeutic potential of EPA and DHA as promising nutritional adjuvants to chemotherapy, which is of particular importance given the prognostic value of myosteatosis as an independent predictive factor of short- and long-term outcomes in cancer. Findings from the present study are novel and encouraging, warranting future research to further delineate mechanisms by which dietary EPA and DHA supplementation attenuate pathological fat accumulation in the skeletal muscles of cancer patients.

## Supporting information

S1 Fig *SREBP-1c* and *C/EBPβ* expression in the rat gastrocnemius muscle. Relative changes in *SREBP-1c* and *C/EBPβ* mRNA expression levels as determined by the 2^−ΔΔCT^ method of analysis. Values are means ± SD.
(DOCX)

S2 Fig Fiber cross-sectional sectional area in the rat gastrocnemius muscle. Values are means ± SD. Different letters indicate significant differences among groups (p<0.05).
(DOCX)

S1 File The ARRIVE guidelines checklist. (PDF)
