# Supplementary material for: Fish oil mitigates myosteatosis and improves chemotherapy efficacy in a preclinical model of colon cancer
Source: PLoS One. 2017 Aug 23;12(8):e0183576. doi: 10.1371/journal.pone.0183576 (PMC5568380; doi:10.1371/journal.pone.0183576)
Supplement: S1 Fig — Relative changes in SREBP-1c and C/EBPβ mRNA expression levels as determined by the 2−ΔΔCT method of analysis. Values are means ± SD. (DOCX) [file pone.0183576.s001.docx]

| **Adjuvant Fish Oil**  **Control**  **Long Term Fish Oil** | **Adjuvant Fish Oil**  **Long Term Fish Oil**  **Control** |
| --- | --- |
