# Supplementary material for: Fish oil mitigates myosteatosis and improves chemotherapy efficacy in a preclinical model of colon cancer
Source: PLoS One. 2017 Aug 23;12(8):e0183576. doi: 10.1371/journal.pone.0183576 (PMC5568380; doi:10.1371/journal.pone.0183576)
Supplement: S2 Fig — Values are means ± SD. Different letters indicate significant differences among groups (p<0.05). (DOCX) [file pone.0183576.s002.docx]

| **Adjuvant Fish Oil**  **Long Term Fish Oil**  **Control**  bc  bc  ab  ab  abb  c  c  bc  a |
| --- |
